# GenomePAM directs PAM characterization and engineering of CRISPR-Cas nucleases using mammalian genome repeats

**DOI:** 10.21203/rs.3.rs-4552906/v1

**Published:** 2025-06-17

**Authors:** Zongli Zheng, Miao Yu, Limei Ai, Bang Wang, Shifeng Lian, James Liu, Linxian Li, Shengdar Tsai, Benjamin Kleinstiver, Lawrence Ip

**Affiliations:** Karolinska Institutet; Karolinska Institutet; Karolinska Institutet; Karolinska Institutet; Karolinska Institutet; GenEditBio; Karolinska Institutet; St. Jude Children’s Research Hospital; Massachusetts General Hospital & Harvard Medical School; GenEditBio Ltd.

## Abstract

Characterizing the protospacer adjacent motif (PAM) requirements of different Cas enzymes is a bottleneck in the discovery of Cas proteins and their engineered variants in mammalian cell contexts. To overcome this challenge and to enable more scalable characterization of PAM preferences, we develop a method named GenomePAM that allows for direct PAM characterization in mammalian cells. GenomePAM leverages genomic repetitive sequences as target sites and does not require protein purification or synthetic oligos. GenomePAM uses a 20-nt protospacer that occurs ~16,942 times in every human diploid cell and is flanked by nearly random sequences. We demonstrate that GenomePAM can accurately characterize the PAM requirement of type II and type V nucleases, including the minimal PAM requirement of the near-PAMless SpRY and extended PAM for CjCas9. Beyond PAM characterization, GenomePAM allows for simultaneous comparison of activities and fidelities among different Cas nucleases on thousands of match and mismatch sites across the genome using a single gRNA and provides insight into the genome-wide chromatin accessibility profiles in different cell types.

## Main Text

In prokaryotes, the CRISPR-Cas system provides antiviral immunity by recognizing and disrupting intruding viral DNA through DNA sequence recognition^1^. This system has been harnessed for precise genome editing in various organisms and cell types^1–5^. Identifying naturally occurring Cas nucleases and engineering Cas enzyme variants with different features is crucial for various research and clinical applications. CRISPR-Cas nucleases form protein:DNA contacts to initiate the target site recognition through a protospacer adjacent motif (PAM)^6–11^. The location (5′ or 3′ of the spacer) and sequence of the PAM differs among different types of CRISPR-Cas systems^11,12^, however, efficient and accurate identification of PAM requirements in eukaryotic cells remains a bottleneck in the discovery and characterization of novel Cas nucleases and their engineered variants.

Various methods have been developed for PAM identification, including in silico^13^, in vitro cleavage assays^12,14–16^, bacterial-based assays^7,10^ including the PAM screen achieved by NOT-gate repression (PAM-SCANR)^17^, cell-free transcription-translation (TXTL) systems^18,19^, fluorescence-based^20^ assays including the PAM definition by observable sequence excision (PAM-DOSE)^21^, human cell library-based approaches^22,23^, and scalable human cell expression followed by *in vitro* cleavage reaction hybrid method high-throughput PAM determination assay (HT-PAMDA)^24,25^. Each method has its own advantages and limitations (reviewed elsewhere^13,25^). In general, in silico and bacterial depletion results may not be easily translated to mammalian cell contexts. In vitro cleavage assays have the advantages of managing large libraries^15^, however, in vitro methods require laborious protein purification, and the cleavage kinetics may not accurately reflect the conditions in a living organism. Previous in vivo methods require introducing synthetic random oligos as PAM candidates into live cells, which are challenging to maintain high-diversity sequence libraries. Also, fluorescence (GFP or RFP)-based enrichments are associated with particularly low efficiency.

Repetitive sequences in the mammalian genome, flanked by diverse sequences, are a potential resource for characterizing the PAM preferences of naturally occurring and engineered Cas nucleases. Here, we developed a direct PAM identification method called GenomePAM, which uses highly repetitive sequences in the mammalian genome. To characterize the PAM requirements of Cas enzymes, we identified genomic repeats flanked by highly diverse sequences where the constant sequence can be used as the protospacer in CRISPR-Cas genome editing experiments. The cleaved genomic regions can then be analyzed using methods such as the Genome-wide Unbiased Identification of Double Strand Breaks (DSBs) Enabled by sequencing (GUIDE-seq)^26^ that enriches double strand oligodeoxynucleotide (dsODN)-integrated fragments by Anchor Multiplex PCR sequencing (AMP-seq)^27^. GenomePAM is highly efficient and accurate at characterizing the mammalian-cell based PAM of an enzyme, because every single cell contains just one full set of identical-complexity candidate PAM library. GenomePAM can also simultaneously assess the potency of thousands of on-target sites across the genome and the fidelity of tens of thousands of potential off-target sites of a Cas nuclease, facilitating performance comparison of different Cas nucleases. Moreover, GenomePAM can be used to better understand and compare genome-wide chromatin accessibility profiles of different cell types.

## Results

### Method design

The human genome contains highly repetitive sequences^28^, most of which are not suitable for use as protospacers due to low-complexity flanking sequences. However, a subset of these sequences can be used for PAM preference identification, provided they have the following features: i) The number of unique flanking sequences of a given length in the human genome is comparable with, or not significantly smaller than, the number of potential PAMs to be tested. For example, the PAM of SpCas9 (*Streptococcus pyogenes* Cas9) and its variants may range from 1 to 3 bases, therefore the number of unique 3-nt long flanking sequences should preferably be 64 (= 4^3^). In the case of a SaCas9^29^(*Staphylococcus aureus* Cas9) and its variants, the PAM may range from 3 to 4 bases, therefore the number of unique flanking sequences of 4-nt long should preferably be about 256 (= 4^4^); and ii) the flanking sequences should have highly diverse or nearly completely random sequence compositions.

**Figure 1:**
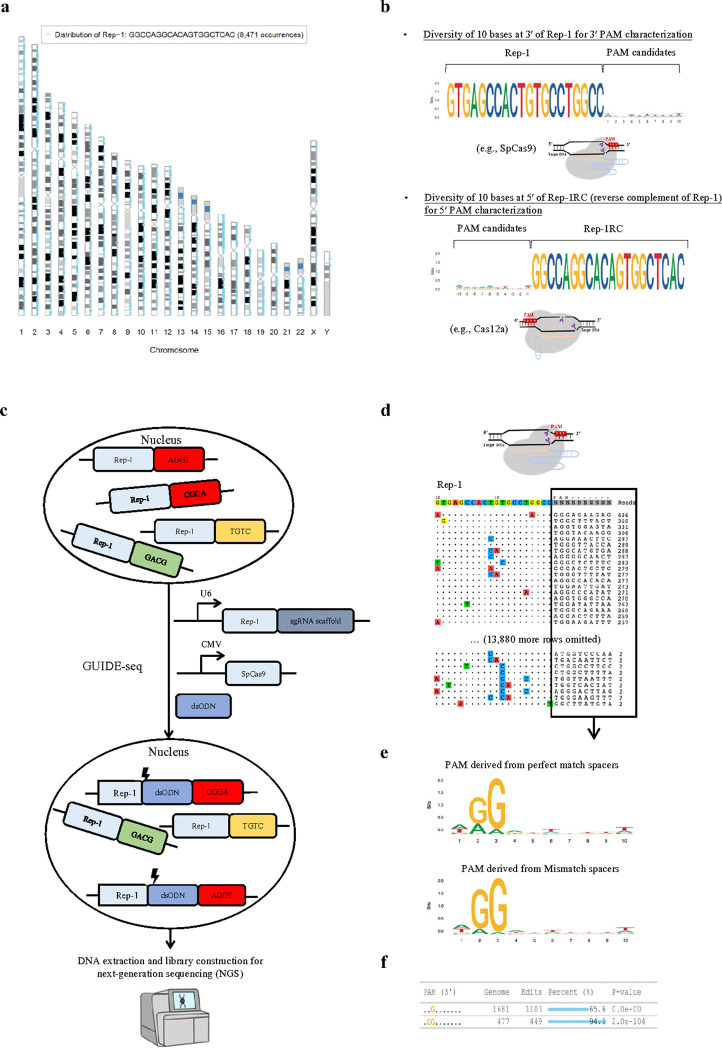
Method design. (**a**) Genome-wide distribution of the Rep-1 sequence in the human genome. (**b**) SeqLogo plot showing nucleotide frequency at each position in the 10 bases at 3′ of Rep-1 and the 10 bases at 5′ of Rep-1RC (reverse complement of Rep-1). Rep-1 and Rep-1RC sequences can be used to characterize PAM preferences of Cas nucleases with 3′ PAM and 5′ PAM, respectively. (**c**) GenomePAM workflow for the identification of PAM preferences using the highly repetitive genome sequence Rep-1 as the protospacer and the GUIDE-seq experiments to capture cleaved genomic sites of SpCas9. (**d**) An example of GUIDE-seq output. Each line showed one SpCas9 cleavage site and the mismatch bases were color coded. The flanking sequences and GUIDE-seq read counts of each site were shown on the right side. (**e**) SeqLogo plot summary for SpCas9 PAM preferences, using their corresponding read counts as weights and stratified by perfect match and mismatch targets. (**f**) GenomePAM Table reported the enriched PAM sequences and counts, along with the numbers and percentages of corresponding genomic sites edited and associated statistical significance. *P*values were derived from *Chi*-square test, two-sided.

To characterize the PAM of SpCas9 and its variants, we analyzed the human genome for all possible 20-nt long sequences and their flanking sequence diversities. For example, there are 8,471 occurrences of the sequence 5′- GTGAGCCACTGTGCCTGGCC −3′ (part of an Alu sequence; hereafter referred to as ‘Rep-1’) distributed across the human genome (Fig. 1a; ~ 16,942 occurrences in a human diploid cell) with nearly random flanking sequences of 10-nt long at its 3′ end, making it a suitable candidate as the protospacer sequence for PAM identification (Fig. 1b). For type II Cas nucleases with 3′ PAMs, such as SpCas9 and SaCas9, Rep-1 can be directly used for PAM preference characterization. For type V Cas nucleases with their PAM at the 5′ end of the spacer, such as FnCas12a^12^ (*Francisella novicida* Cas12a), the reverse complementary sequence 5′- GGCCAGGCACAGTGGCTCAC-3′ (‘Rep-1RC’) can be used as the protospacer sequence (Fig. 1b). Since Cas nucleases can often tolerate a few base mismatches (off-targets), we calculated the numbers of 20-nt sequences with 1, 2, 3, and 4 base mismatches. For Rep-1, these numbers were 48,207, 206,767, 579,336, and 1,350,488, respectively, and > 2 million in total in the human genome (hg38). Thus, using Rep-1 or Rep-1RC as the protospacer, there are potentially > 4 million targets in one single human diploid cell. A list of example repeats, their occurrences, flanking sequence diversity and their use are shown in **Extended Data Fig. 1**.

To leverage these genomic repeats to characterize the PAM of various CRISPR-Cas enzymes, the repeat sequence Rep-1 was chosen as the protospacer target. The corresponding spacer was cloned into a gRNA expression cassette to be used along with a plasmid that encodes the candidate Cas nuclease. To identify which repeats within the genome were cleaved in an experiment, we adapted the genome-wide, unbiased identification of DSBs enabled by sequencing (GUIDE-seq^26^) method to capture cleaved genomic sites in HEK 293T cells (Fig. 1c). Only those sites whose flanking sequences contain functional PAMs can be cleaved by the Cas nuclease. Cell toxicity after large numbers of DSBs occur in one cell were reported previously when using CRISPR to target highly repetitive element LINE1^30^ or unique repeat sequences associated with temozolomide mutational signature^31^. To access this toxicity, we measured cell viability in four different conditions, including Lipofectamine 3000 transfection controls, and in two different cell lines (see Methods). The results showed largely similar cell viability across different transfection conditions at 24 h and 48 h after transfection in HEK293T (**Extended Data Fig. 2a**) and HepG2 cells (**Extended Data Fig. 2b**). During the GUIDE-seq data analysis, the candidate PAM was set as unknown (“NNNNNNNNNN”) and 13,908 sites across the genome were identified (Fig. 1d). The mismatch bases were typically located at positions 8 to 11 of the targets and were transitions of the intended bases (Fig. 1d). The resulting PAMs were then summarized using their corresponding read counts as weights and used for SeqLogo plotting (Fig. 1e), which was stratified by perfect-match and mismatch targets. Beyond the descriptive SeqLogo, the consistent genomic background sequences inspired us to create an iterative ‘seed-extension’ method. This approach identifies statistically significant enriched motifs and reports the percentages of edited genomic sites at each iteration step. (Fig. 1f; See Methods).

### Performance of GenomePAM on SpCas9, SaCas9 and FnCas12a

**Figure 2:**
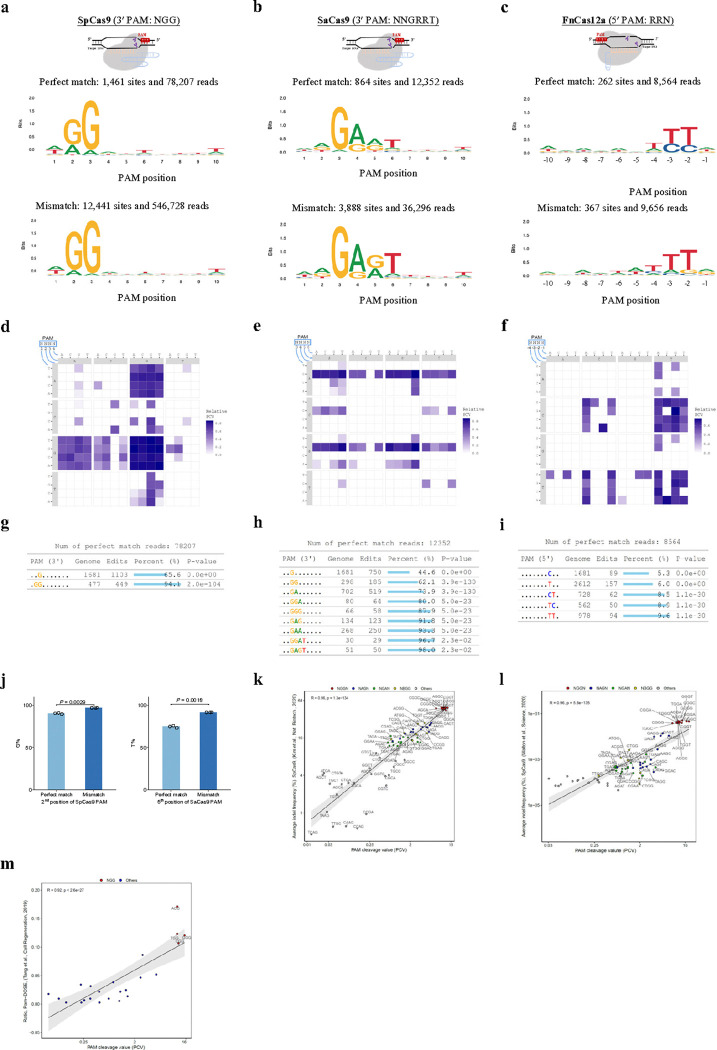
Evaluation of the GenomePAM assay on the PAM characterization for SpCas9, SaCas9 and FnCas12a. SeqLogo results for SpCas9, SaCas9 and FnCas12a PAM preferences in HEK293T cells with perfect match spacers (**a**,**b**, and **c: top**), with mismatch spacers (**a**, **b**, and **c: bottom**), and plotted in a 4-base heatmap of relative PAM cleavage value (PCV) (**d**, **e**, and **f**) and corresponding GenomePAM Tables (**g**, **h**, and **i**). *P*values were derived from *Chi*-square test, two-sided. (**j**) Percentage of ‘G’ at the 2nd position of SpCas9 PAM (**left**) and percentage of ‘T’ at the 6th position of SaCas9 PAM (**right**), by cleaved target types (perfect match vs mismatch). Data are presented as mean values +/− SD. Correlations between PCV of GenomePAM with indel frequencies reported by previous methods, Kim et al, Nat. Biotech. 2020 (**k**), HT-PAMDA (**l**) and PAM-DOSE (**m**), across various SpCas9 PAM sequences, including canonical (NGGN) and non-canonical PAMs (NAGN, NGAN, NBGG; ‘B’ is C, G, or T). Linear regression lines were plotted, with 95% confidence intervals indicated as the grey areas.

To evaluate the performance of GenomePAM, we chose three Cas nucleases with well-established PAMs: SpCas9, SaCas9, and FnCas12a. We used Rep-1 for GenomePAM analysis of SpCas9 and SaCas9, and Rep-1RC for FnCas12a. The results showed that the PAM preferences for SpCas9, SaCas9 and FnCas12a were NGG at 3′, NNGRRT (R is G or A) at 3′ and TTTN at 5′ sides of the spacers, respectively, consistent with previous results^7,10,12,29,32,33^ (Fig. 2a,b,c; **top**, perfect match; **middle**, mismatch; **bottom**, 4-base heatmap of relative PAM cleavage value (PCV), see Methods; GUIDE-seq results were listed (**Supplementary Tables 1 – 3**) and visualized (**Supplementary Tables 4 – 6**). Relative PAM cleavage values of the three nucleases are visualized in heatmaps (Fig. 2d,e,f). GenomePAM Table analysis showed that, for SpCas9 3′ PAM, the most significant single base was the G at position 3 (1,103 (65.6%) of total 1,681 targets in human genome edited), the most significant two bases were GG at positions 2 and 3 (449 (94.1%) out of total 477 targets edited), and no further significant bases (Fig. 2g). For SaCas9 3′ PAM, the GenomePAM Table analysis showed increasing significance for G at position 3, GR at 3 to 4, GRR at 3 to 5, and GRRT at 3 to 6, respectively (Fig. 2h). The corresponding percentages of edited targets were 44.6%, 62.1% to 73.9%, 80.0% to 93.3%, and 96.7% to 98.0%, respectively (Fig. 2i). For FnCas12a 5′ PAM, the GenomePAM Table analysis showed a Y position −3 (5.3% to 6.0% of targets edited) and a YY at position −2 to −3 (8.5% to 9.6% of targets edited). For the known PAM wobble bases^6,34^, namely the 2^nd^ position of N[G/A]G in SpCas9 and the 6^th^ position of NNGRR[T/A] in SaCas9, the proportions of the prominent bases ([G] in SpCas9 and [T] in SaCas9) in the mismatch target-associated PAMs were higher than those perfect match-associated PAMs (Fig. 2j; **left**, SpCas9; **right**, SaCas9; both *P* < 0.01). We also performed validation experiments in three other cell lines, HepG2, Huh7, and HeLa cell lines (**Extended Data Fig. 3**). The results showed nearly identical PAM profiles as those using HEK 293T cells. Furthermore, we tested using another repeat sequence (Rep-2: 5′- GAGCCACCGTGCCTGGCCTC −3′) that occurs 1,126 times in the human genome (~ 2,252 occurrences in a human diploid cell) (**Extended Data Fig. 1**) as the protospacer for GenomePAM analysis of SpCas9, SaCas9, and FnCas12a. The GenomePAM results were nearly the same, namely NGG at 3′, NNGRRT at 3′, and TTTN at 5′ for the three nucleases, respectively (**Extended Data Fig. 4**). We further compared SpCas9 canonical (NGGN) and non-canonical PAM (NAGN, NGAN, NBGG; ‘B’ is C, G, or T) PCVs derived from GenomePAM with indel frequencies reported by three well-established methods^21,22^,24. The analyses showed high correlations (Fig. 2k,l, vs two assays: R = 0.96, *P* < 1 × 10^−100^; Fig. 2m, vs PAM-DOSE: R = 0.92, P < 2.6 × 10^−27^). Our results recapitulate the known PAM requirements of type II and type V Cas nucleases, demonstrating that our genome-based PAM determination method (GenomePAM) is effective.

### Characterization of challenging PAM preferences

**Figure 3:**
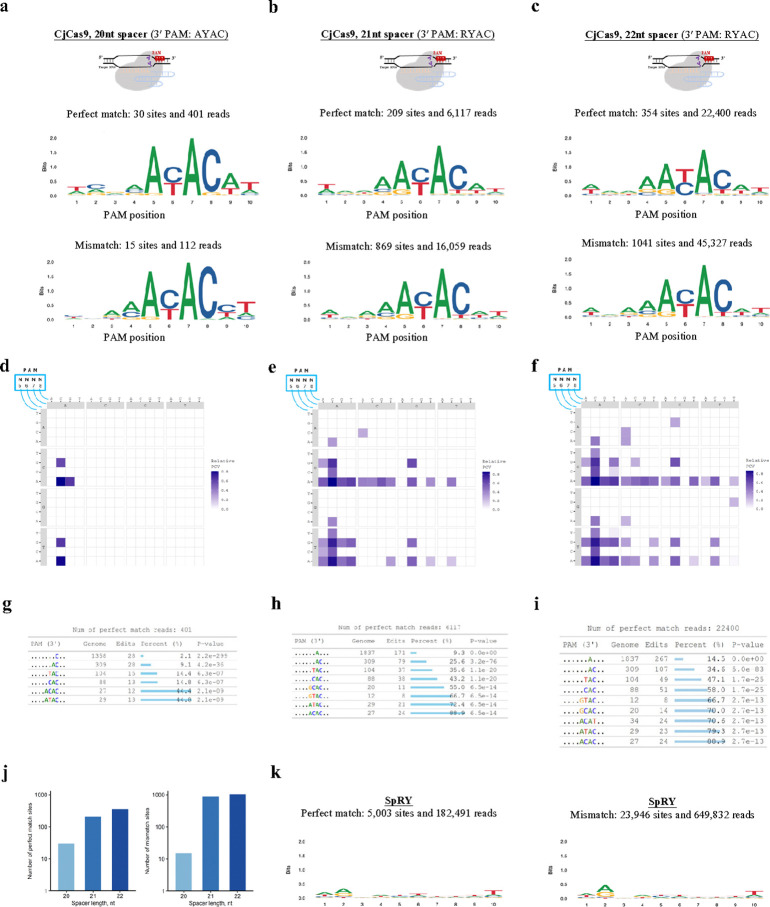
Evaluation of the GenomePAM assay on the PAM characterization for CjCas9 and SpRY. SeqLogo results for CjCas9 when using different lengths of spacers: (**a**) 20 bases (‘GTGAGCCACTGTGCCTGGCC’), (**b**) 21 bases (‘YGTGAGCCACTGTGCCTGGCC’, ‘Y’ is ‘C’ or ‘T’), and (**c**) 22 bases (‘GYGTGAGCCACTGTGCCTGGCC’) with perfect match cleaved sites (**top**) in HEK293T cells and in their mismatch cleaved sites (**bottom**); and corresponding 4-base heatmap of relative PAM cleavage values (PCVs) (**d**, **e**, **f**) and GenomePAM Tables (**g**, **h**, **i**). *P*values were derived from *Chi*-square test, two-sided. (**j**) The numbers of perfect match sites (**left**) and mismatch sites (**right**) cleaved by CjCas9 using 20 nt, 21 nt and 22 nt spacers, respectively. SeqLogo results for SpRY PAM preferences in HEK293T cells with perfect match spacers (**k, left**) and mismatch spacers (**k, right**).

Long and complicated PAMs in naturally occurring Cas nucleases pose a challenge to identifying their PAMs experimentally. We evaluated the performance of GenomePAM on *Campylobacter jejuni* Cas9 (CjCas9), which was reported to require a 7-nt PAM NNNNACA^35^ and an 8-nt one NNNNRYAC^36^. We performed GenomePAM assay on CjCas9 using Rep-1 as the protospacer in HEK 293T cells. The results showed that CjCas9 required NNNNRYAC as its PAM in HEK 293T cells (Fig. 3a; **top**, perfect match; **bottom**, mismatch). Because the optimal length of protospacer for CjCas9 was shown to be 22 bases^36^, we tested using extended Rep-1 to 21 (5′ YGTGAGCCACTGTGCCTGGCC 3′, Y is C or T) and 22 (5′ GYGTGAGCCACTGTGCCTGGCC 3′) bases. The results showed nearly the same NNNNRYAC PAM preferences for both 21 and 22 base protospacers (Fig. 3b,c; **top**, perfect match; **bottom**, mismatch). Relative PAM cleavage values using these protospacers are visualized in heatmaps (Fig. 3d,e,f). GenomePAM Table analysis consistently showed the most enriched sequence ACAC at positions 5 to 8 (Fig. 3g,h,i). The longer protospacers with 21 and 22 bases showed marked increases in cleavage activities compared with the 20-base spacer for CjCas9 (Fig. 3j). The PAM requirement on the 8^th^ position was relatively relaxed (Fig. 3b, c), and there were increases in the numbers of off-target sites (Fig. 3a,b,c; **bottom**).

Another challenging scenario in characterizing PAM preference is when there is little preference. Engineering Cas nucleases to relax PAM requirements can broaden potential applications, such as using the SpRY variant of SpCas9 with nearly no PAM restriction (previously described to be NRN>NYN)^24^. Additional near PAMless Cas variants have also been developed^37,38^. However, depletion-based methods may not be efficient in identifying Cas nucleases with nearly no PAM preferences^25^. Being a positive selection method, GenomePAM found that as expected, SpRY exhibited a very minimal PAM requirement, being nearly PAMless across 5,003 perfect match loci and 23,946 mismatch loci in HEK 293T cells (Fig. 3k).

### GenomePAM for characterizing novel Cas PAM

**Figure 4:**
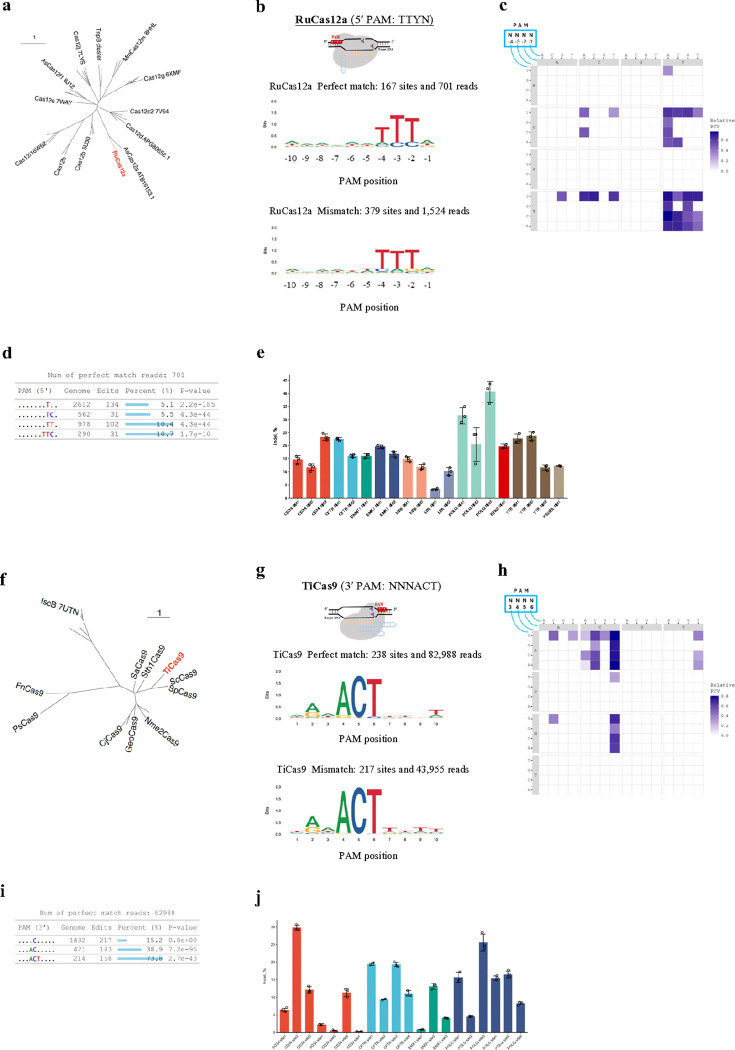
Novel Cas nuclease discovery and their PAM identifications using the GenomePAM assay. (**a**) Phylogenetic tree for the Type V-A Cas system, RuCas12a. (**b**) SeqLogo results for RuCas12a PAM preferences in HEK293T cells with perfect match spacers (**left**) and mismatch spacers (**right**), associated 4-base heatmap of relative PAM cleavage value (PCV) (**c**), and the GenomePAM Table (**d**). *P* values were derived from *Chi*-square test, two-sided. (**e**) Editing efficiency of RuCas12a on 20 regular genomic sites in human genes *CD34, CFTR, DNMT1, EMX1, HBB, LPA, POLQ, RFN2, TTR* and *VEGFA* with a 5′-TTTG PAM. Data are presented as mean values +/− SD. (**f**) Phylogenetic tree for the Type II CRISPR-Cas system, TiCas9. (**g**) SeqLogo results for TiCas9 PAM preferences in HEK293T cells with perfect match spacers (**left**) and mismatch spacers (**right**), a 4-base heatmap of relative PAM cleavage value (PCV) (**h**), and the GenomePAM Table (**i**). *P* values were derived from *Chi*-square test, two-sided. (**j**) Editing efficiency of TiCas9 on 20 regular genomic sites in human genes *CD34, CTCF, EMX1, POLQ* and *VEGFA*with a 3′-NNNACT PAM. Data are presented as mean values +/− SD.

After establishing the simplicity and accuracy of the GenomePAM assay in identifying various Cas nucleases, we sought to demonstrate its utility in PAM identification for novel Cas discovery. Using a metagenomics approach (see Methods) to analyze recent data in NCBI Sequence Read Archive (SRA), we identified one novel type V-A CRISPR-Cas candidate derived from *Ruminococcus Dsp902787825*, named RuCas12a (Fig. 4a). We performed the GenomePAM assay using ‘Rep-1’ as the protospacer to characterize its potential activity and 5′ PAM requirement. The result revealed that the PAM preference of RuCas12a was TTYN at its 5′ end (Fig. 4b,c). GenomePAM Table showed dominant TTC at positions −4 to −2 (Fig. 4d, the bottom row). To further validate and comprehensively evaluate genome editing efficiency of RuCas12a, we used 20 regular (non-repetitive) genomic sites containing a 5′ end ‘TTTG’ in human genes *CD34, CFTR, DNMT1, EMX1, HBB, LPA, POLQ, RFN2, TTR* and *VEGFA* (spacer and primer sequences are listed in **Supplementary Table 7)**. The editing efficiencies ranged from 3.4% to 40.6% across the 20 genomic loci in HEK293T cells (Fig. 4e). We also applied GenomePAM for identifying PAM of novel type II Cas nuclease and found a novel Cas9 from *Tissierella* sp., named TiCas9. TiCas9 clusters closely to SpCas9 and ScCas9, implying that it is a type II-A Cas nuclease (Fig. 4f). GenomePAM analysis revealed that TiCas9 had a NNNACT PAM (Fig. 4g,h,i). We further validated its potencies across 20 endogenous loci with a 3′-NNNACT PAM in genes *CD34, CTCF, EMX1, POLQ* and *VEGFA* in HEK293T cells, which showed up to ~ 30% editing efficiency using its native gRNA scaffold (Fig. 4j; Spacer and primer sequences are listed in **Supplementary Table 8**).

### GenomePAM facilitates Cas PAM engineering

**Figure 5:**
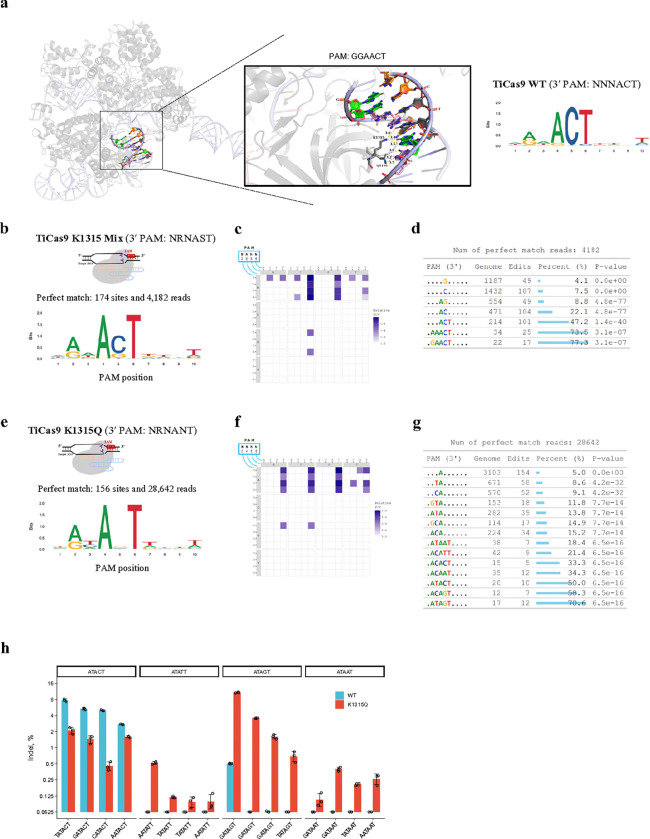
Structure-guided engineering for altered PAM preference of TiCas9. The structure of TiCas9 and sgRNA complex was predicted by using AlphaFold 3 and visualized in PyMOL 3.0 (**a**). The PAM-interacting domain containing polar residue-DNA interactions within 4 Å are shown. Hydrogen bonds between K1315 or Q1191 and nitrogenous base in the anti-sense strand of PAM were indicated by yellow dash line, with corresponding distances labeled. SeqLogo shows the GenomePAM result of TiCas9-WT. (**b**) SeqLogo visualization of the 3′ PAM preference of the pooled NNK library of the TiCas9 1315 variants; (**c**) the associated 4-base heatmap of relative PAM cleavage value (PCV); and (**d**) the GenomePAM Table. *P*values were derived from *Chi*-square test, two-sided. (**e**) SeqLogo visualization of the 3′ PAM preference of the TiCas9 K1315Q variant; (**f**) the associated 4-base heatmap of relative PAM cleavage value (PCV); and (**g**) the GenomePAM Table. *P* values were derived from *Chi*-square test, two-sided. (**h**) Indel percentages using TiCas9 WT and its K1315Q variant on sixteen endogenous sites in *RNF2* harboring different PAM sequences (positions 4 to 6: ACT, ATT, AGT, and AAT). Data are presented as mean values +/− SD.

Engineering Cas PAM preference to expand the targetability represents an attractive strategy for broad applications^38^. To this end, we questioned whether GenomePAM could facilitate the Cas variant discovery. We showed here using TiCas9 as an example by first applying GenomePAM to profile pooled mutant variants and, upon evidence of altered mixed PAMs, followed by GenomePAM characterization of single-mutant variants (Fig. 5). Because there are many Cas9 nucleases recognizing G/C-rich PAM, we aimed to engineer TiCas9 for recognizing A/T-rich PAM, namely to relax the C at position 5 of NNNACT. Using AlphaFold 3^39^, we identified that K1315 was the only residue found to interact with Gat position 5 on the complementary strand (Fig. 5a). We constructed an NNK library encoding for all 20 amino acids at position 1315. GenomePAM analysis of the pooled variants showed dramatically altered base compositions at position 5, without affecting positions 4 and 6, in the aggregated PAMs (Fig. 5b,c,d). We then assessed all the 19 a.a. variants individually. The variant K1315Q showed completely no restriction at position 5 (Fig. 5e,f,g), namely an ANT PAM at positions 4 to 6, while other 18 variants showed varied preferences at position 5 (**Extended Data Fig. 5**). Then, sixteen endogenous sites in *RNF2* harboring PAM positions 4 to 6 (4 ACT, 4 ATT, 4 AGT, and 4 AAT) were used to validate the variant K1315Q vs WT (spacer and primer sequences are listed in **Supplementary Table 9**). The results were consistent with SeqLogo, PCV visualization, and GenomePAM Table (Fig. 5h). Interestingly, GenomePAM Table showed that, for PAM positions 4 to 6, the proportions of genome-wide target sites edited were highest with AGT, followed by ACT, and the lowest with AAT and ATT – largely consistent with the indel percentages at the 16 endogenous sites tested individually (Fig. 5g vs 5h).

### Comparison of genome-wide potency and specificity

**Figure 6:**
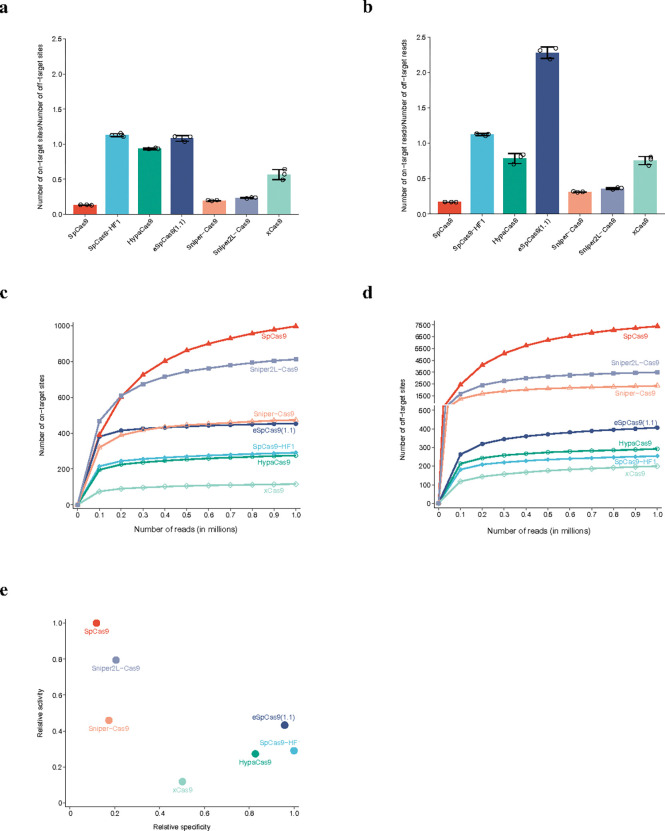
General activities and specificities of different SpCas9 variants. (**a**) The number of on-target sites to the number of off-target sites of seven SpCas9 variants. Data are presented as mean values +/− SD. (**b**) The number of on-target sequencing reads to the number of off-target sequencing reads of seven SpCas9 variants. Data are presented as mean values +/− SD. The number of on-target sites (**c**) and the number of off-target sites (**d**) detected when using randomly down-sampled datasets, from 0.1 M to 1 M raw sequencing reads. (**e**) Relative activities (defined as the number of perfect match sites relative to that of the WT SpCas9) and specificities (defined as the ratio of perfect match to mismatch site numbers relative to the ratio in SpCas9-HF1) of seven SpCas9 variants.

Many SpCas9 variants have been developed to reduce off-target effects, such as SpCas9-HF1^40^, HypaCas9^41^, eSpCas9(1.1)^42^, Sniper-Cas9^43^, and Sniper2L-Cas9^44^ and broadened PAM compatibilities like xCas9^45^. Oftentimes, dozens of gene loci are used to assess the fidelity and activity of Cas9 variants^41,43^. It would be desirable to simultaneously evaluate Cas nuclease potency and specificity with a less laborious method than traditional library-based approaches^22,46^. A method based on large-scale synthetic oligos (n = 26,891) containing targeting sequences and mismatch sequences has been developed for this purpose^22^. GenomePAM uses a single protospacer oligo that provides thousands of perfect match sites and millions of mismatch sites in one human cell. Therefore, we sought to evaluate the feasibility of simultaneously comparing genome-wide potency and specificity of different Cas9 variants. We performed GenomePAM experiments with ‘Rep-1’ as the targeting protospacer for WT SpCas9 and seven variants in parallel, six of which were compared in a previous study (SpCas9-HF1, eSpCas9(1.1), HypaCas9, xCas9, and Sniper-SpCas9) and the same group developed a new variant (Sniper2L-SpCas9), with the same amounts of Cas and of sgRNA expression plasmids (**Extended Data Fig. 6**). The ratios of on-to-off target sites were highest for SpCas9-HF1 (mean 1.13), eSpCas9(1.1) (mean 1.08), and HypaCas9 (mean 0.93), followed by xCas9 (mean 0.57), Sniper2L-Cas9 (mean 0.23) and Sniper-Cas9 (mean 0.20), and the lowest for WT SpCas9 (mean 0.13) (Fig. 6a). Similarly, the ratios of on-to-off target reads were 1.12, 2.28, 0.78, 0.75, 0.36, 0.31 and 0.17, respectively (Fig. 6b). To evaluate Cas9 cleavage dynamics with increasing probing data, we sampled the datasets from 100K up to 1M sequencing reads for GUIDE-seq analysis. The number of on-target sites identified given the same amount of sequencing data was the highest (the most potent) in WT, followed by Sniper2L-SpCas9, and then comparably in Sniper-SpCas9 and eSpCas9(1.1), and the lowest in SpCas9-HF1, HypaCas9 and xCas9 (Fig. 6c). The numbers of off-target sites identified given the same amount of data were the lowest (the most specific) in xCas9, HypaCas9, SpCas9-HF1, and eSpCas9(1.1), and then comparably in Sniper-SpCas9 and Sniper2L-SpCas9, and the highest (the least specific) in WT (Fig. 6d).

To compare general activity and specificity of different SpCas9 variants in one place, we used the 1M-read datasets. We defined relative activity as the number of perfect match sites relative to the number of perfect match sites identified by WT SpCas9, and relative specificity as the ratio of perfect match to mismatch target site numbers relative to the same ratio in SpCas9-HF1 (because SpCas9-HF1 had the highest ratio among the seven SpCas9 tested here) (Fig. 6e) The scatter plot showed that WT and Sniper2L- SpCas9s were more potent but less specific than other variants, whereas eSpCas9, SpCas9-HF1 and HypaCas9 were more specific but less potent than WT SpCas9 (Fig. 6e). To evaluate whether using a different repetitive spacer for GenomePAM can affect the general activity and specificity, we used Rep-3 (**Extended Data Fig. 1**) and the results were similar to those obtained with Rep-1 (**Extended Data Figs. 7,8**).

### Profiling chromatin accessibility in different human cell lines.

**Figure 7:**
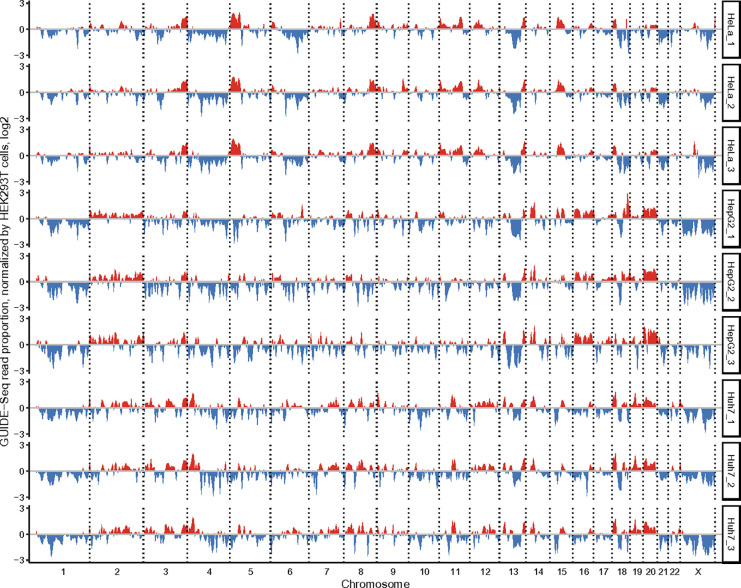
The GenomePAM assay profiles of chromosome accessibility when using SpCas9 and targeting ‘Rep-1’ in human cell lines HeLa, HepG2, and Huh7. The proportion of cleavage read counts in each five-million base chromosomal window was divided by the proportion in the corresponding chromosomal window in the HEK293T cells (mean of triplicates) and log2-transformed. A higher cleavage proportion relative to HEK293T was colored in red and a relatively lower proportion in blue.

Chromatin conformation has been shown to affect Cas nuclease genome editing^47,48^ on relatively small numbers of genomic targets but not on the genome-wide scale. To assess genome-wide targeting profiles in different cell lines, we performed GenomePAM assays using SpCas9 and ‘Rep-1’ in HEK293T, HepG2, Huh7 and HeLa cell lines with triplicates (Fig. 7). Genome-wide chromatin accessibility was defined as the number of targeting reads per 5 M base chromosome window. Relative to HEK293T, the results from triplicates of the same cell line showed consistent and reproducible genome-wide chromatin profiles (**Extended Data Fig. 9**). Interestingly, hepatocyte-derived cell lines HepG2 and Huh7 showed very similar chromatin accessibility profiles in contrast to that of HeLa cells, indicating similar chromatin accessibility in the same tissue (Fig. 7).

## Discussion

In this study, we developed a new method called GenomePAM and demonstrated its simplicity, accuracy and capability in assaying PAM preferences of previously established SpCas9, SaCas9 and FnCas12a nucleases, as well as complicated and challenging PAM recognitions as in SpRY and CjCas9. We also demonstrated the potential of GenomePAM for simultaneous comparison of potencies (thousands of perfectly matched loci) and, when combined with GUIDE-seq, fidelities (tens of thousands of off-target sites) of various Cas nucleases and variants. Compared with other PAM identification methods using regular PCR amplicon sequencing, GenomePAM uses GUIDE-seq and thus requires relatively more skills to perform. However, GUIDE-seq has been one of the main methods for assessing CRISPR off-target effects in both research and therapeutic settings^49,50^. Using GenomePAM, we rapidly identified one type II Cas TiCas9 and one type V Cas RuCas12a that are both active in human cells. Directed by AlphaFold 3, we further accelerated the PAM engineering of TiCas9 to expand its targetability using GenomePAM. Additionally, we demonstrated at the genome-scale that genomic accessibility of a given CRISPR-Cas design differs among cells of different tissue types. We envision that GenomePAM will be widely useful for discovery, characterization, and comprehensive evaluation of the PAM recognition, potency, and fidelity of CRISPR-Cas nucleases and engineered variants.

GenomePAM is capable of direct identification of challenging PAMs in human cells. Different PAM preferences for CjCas9 have been reported. An in-silico prediction followed by a biochemical digestion assay reported that the PAM for CjCas9 was NNNNACA^35^, while an in vitro cleavage assay followed by in vivo second-step analyses on each of the positions from 5 to 8 showed that the optimal PAM was NNNNRYAC^36^. Maintaining large-scale libraries consisting of many sequences is challenging. Previous methods have attempted to address this by using sequential rounds of experiments in exceptionally challenging situations, with progressively lengthened PAM candidate sequences^12,14^. Maintaining rich library complexities in large-scale screening experiments is often challenging, however, is not an issue in GenomePAM since every single cell contains one full set of potential PAM candidates, maintaining PAM candidate sequence diversity is not an issue in GenomePAM. We used GenomePAM in a one-round experiment directly in human cells and showed that, without prior protein purification and without introducing a library of synthetic oligos, the PAM preference of CjCas9 was NNNNRYAC. Since GenomePAM is a positive selection method, it can be used to efficiently identify PAM requirements when there are no preferences^25^.

Methods that can compare potency and fidelity of various Cas nucleases simultaneously are highly desirable. One such method involves constructing stable cell lines with balanced expression of Cas nucleases and variants to be compared, followed by transduction of a large pool of synthetic oligos (n = 26,891, on- and off-target sequences) at a carefully controlled multiplicity of infection (MOI) into these stable expression cells to compare potency and fidelity of various Cas nucleases^22^. One advantage of this approach is that it includes different on-target and off-target sequences. GenomePAM takes advantage of highly repetitive sequences in every cell (thousands of on-targets and tens of thousands of off-target sequences), is much simpler and of low cost. One limitation of GenomePAM is that it uses relatively limited kinds of on-target sequences, although they appear thousands of times in one cell. However, this can be compensated by using different repetitive sequences, such as Rep-1, Rep-3, or Rep-4 for result confirmation and validation, and in different cell types. Indeed, our results showed that using different sequences (Rep-1, Rep-2 or Rep-3) as the protospacers for GenomePAM analyses gave the same results in PAM characterizations for the different Cas nucleases tested in this study. However, a new Cas nuclease might have a scaffold sequence that interferes with the repeats, potentially forming strong secondary structures and affecting GenomePAM results. We recommend using at least two different repeats as GenomePAM spacers for novel Cas nucleases. Another possibility is to combine different repetitive sequences in one experiment, although we have not tested this ourselves yet. In such a case, bioinformatic analysis would need to use one repetitive sequence at a time and repeat the data analysis for all sequences. Chromatin accessibility affects Cas nuclease activity, as shown on a genome-wide scale. The GenomePAM assay is minimally biased by chromatin accessibility, likely due to the large number of accessible perfect-match targets in each cell.

Measuring chromatin accessibility is important in understanding basic cellular processes, including transcription, replication, chromosome segregation, and DNA repair^51^. A variety of techniques like Dnase-seq^52^ and ATAC-seq^53,54^ enable quantifying genome-wide chromatin accessibility. Genome accessibility to CRISPR-Cas targeting is known to differ among different cell types but has been demonstrated only in limited and selected loci. GenomePAM demonstrates clearly that the CRISPR-Cas genome-wide accessibility profiles differ among different cell types. GenomePAM may complement existing methods for studying genome-wide chromatin dynamics.

## Methods

### Identification of repeat sequences

The human genome (hg38) was used to calculate the frequencies of all 20-mer sequences using the jellyfish tools^55^. Because a spacer starting with a G base at 5′ is required for most Cas nucleases, we selected all 20-mer sequences starting with a 5′ G. To avoid simple homopolymers and to increase base composition diversity, we also excluded those 20-mers containing ‘AAA’, ‘TTT’, ‘CCC’, or ‘GGG’. Among the remaining 20-mer sequences, to retrieve their flanking sequences, we used BWA^56^ to map their chromosomal coordinates and retrieved 10 bases upstream and 10 bases downstream using samtools^57^. The diversity of the flanking sequences of each of the 20-mer sequence was plotted using the ggseqlogo^58^ package. We define PAM cleavage value (PCV) as the ratio of the percentage of a given PAM sequence among all sequences of the same length captured by GenomePAM to the percentage of the same PAM sequence among all sequences of the same length in the human genome (hg38). To better visualize the PAM recognition pattern in 4-base heatmap, a relative PCV was calculated by taking log2 transformation of PCV and normalized to the PAM sequence with the highest PCV.

### CRISPR/Cas identification

Metagenomes were downloaded from EMBL-EBI MGnify, NCBI GenBank and the Joint Genome Institute, or assembled in-house using raw sequencing reads from NCBI Sequence Read Archive. We used a combinatorial pipeline that includes CCTyper^59^, CRISPRcasIdentifier^60^ and OPFI^61^ to predict putative Cas proteins. MinCED^62^ was used to identify CRISPR arrays, any CRISPR arrays located adjacent to the predicted Cas, which typically comprises a CRISPR operon, were subjected to further analysis. Putative sequences of Cas were scanned by Interproscan^63^ to identify and annotate conserved domains. Selected Cas proteins were aligned with MAFFT^64^, and a phylogenetic tree was constructed using FastTree2^65^.

### Cell culture

HEK293T (CRL3216, ATCC), HepG2 (CRL11997, ATCC) and HeLa (CCL-2, ATCC) cell lines were purchased from ATCC (American Type Culture Collection, VA, USA). Huh7 (01042712, Sigma) cell line was purchased from Sigma. HEK293T cells, HeLa cells and Huh7 cells were cultured in Dulbecco’s Modified Eagle Medium (C11995500BT, GIBCO), HepG2 cells were cultured in Eagle’s Minimum Essential Medium (30–2003, ATCC) supplemented with 10% fetal bovine serum (10270–106, GIBCO) and incubated at 37 °C with 5% CO_2_ in a constant temperature incubator. Cell passaging was performed at a 1:3 split ratio when the cells reached 90% confluence.

### Plasmids and oligonucleotides

The plasmids used in these experiments were purchased from the non-profit plasmid repository Addgene (Watertown, MA, U.S.). The plasmid lentiCRISPRv2 (Addgene #52961) was used to express wild-type SpCas9; BPK2139 (Addgene #65776) to express wild-type SaCas9; pY004 (Addgene #69976) to express wild-type FnCas12a; pET-CjCas9 (Addgene #89754) to express wild-type CjCas9 and the plasmids BPK1520 (Addgene #65777), BPK2660 (Addgene #70709), pU6-Fn-crRNA (Addgene #78958), pU6-cj-E sgRNA (Addgene #169915) were used to express SpCas9 sgRNA, SaCas9 sgRNA, FnCas12a crRNA and CjCas9 sgRNA, respectively. Oligonucleotide duplexes corresponding to the target spacer sequences were purchased from GENEWIZ (Suzhou, China).

### Plasmid construction

Oligonucleotide duplexes corresponding to sgRNA sequences (paired top and bottom single-stranded oligos) were annealed together using the following program: 95°C, 3min; 70 cycles of (95°C, 1 min with −1°C per cycle); 4°C hold. Annealed DNA segments were inserted into BsmbI digested sgRNA expression plasmids. After transformation into bacteria and selection, the plasmids were purified by PureLinkTM HiPure Plasmid Midiprep Kit (Invitrogen). Sequences of guide-insertion in the plasmids were confirmed by Sanger sequencing (BGI, Hong Kong)

### dsODN preparation

dsODN oligos were purchased from GENEWIZ with HPLC purification. Each oligo was resuspended in 1×TE buffer (Thermo Fisher 12090015) to a final concentration of 250 μM. These oligos were then annealed at 100 μM in 1× annealing buffer (10mM Tris-HCl, 50mM NaCl, 1mM EDTA, pH7.4) on thermocycler. The program was 95°C, 3min; 70× [95°C 1 min (−1°C per cycle)]; 4°C hold.

### Cell transfection

Guide RNAs and Cas proteins plasmids were transfected into cells using Lipofectamine^™^ 3000 Transfection Reagent (ThermoFisher, L3000015) following the manufacturer’s instruction. Cells were cultured at the density of 1 × 10^5^ per well in 24-well plate. For each well, 100 ng of gRNA and 400ng of Cas expression plasmids together with 5 pmol of annealed dsODN were mixed with 1 μL of P3000 reagents in 25 μL of Opti-MEM medium, and then mixed with 1.5 μL of Lipofectamine 3000 Reagent in 25 μL of Opti-MEM medium to generate a total volume of 50 μL DNA-lipid complex, followed by incubation for 10 minutes at room temperature. The transfection complex was added into individual wells. The plate was maintained in a cell culture incubator for 48–72 hours.

### Cell viability assay

HEK293T and HepG2 cells were seeded in 96-well plates at 2.0 × 10^4^ per well and transfected with four different conditions: (1) SpCas9 plasmid + Rep-1 sgRNA plasmid + dsODN; (2) SpCas9 plasmid + Rep-1 sgRNA plasmid; (3) SpCas9 plasmid + non-targeting sgRNA plasmid + dsODN; and (4) Lipofectamine 3000 only. The cell viability was performed with Enhanced Cell Counting Kit-8 (Beyotime, C0043) according to the manufacturer’s instruction. Briefly, 10 μL CCK-8 labeling reagent was added to each well and incubated at 37°C with 5% CO2 in a humidified atmosphere for 1 hour. Cell viability was then determined by the absorbance at 450 nm and evaluated 0 h, 24 h and 48 h after transfection.

### DNA extraction

Genomic DNA was extracted using the MiniBEST Universal Genomic DNA Extraction Kit (TaKaRa) and quantified using QubitTM dsDNA HS Assay Kit (Invitrogen) in Qubit 3.0 fluorometer.

### GUIDE-seq

Genomic DNA was extracted 48–72 hours post-transfection and 500 ng of DNA was used for next-generation sequencing (NGS) library construction according to our previous work^26^ with modification^66^ (see updated dsODN sequences and amplification primers with discussion notes in **Supplementary Table 10**). Briefly, the library preparation procedure contains enzymatic fragmentation, end-repair, A-tailing, adaptor ligation and two rounds of anchored nested PCR. The libraries were quantified with KAPA Library Quantification Kits and sequenced on NextSeq 1000 System (Illumina, San Diego, CA, USA) using 300-cycle kit (2×150bp paired-end) with standard Illumina sequencing workflow, namely no need to adjust indexing cycles nor the need to use customized sequencing primers. Sequencing data (FASTQ files) were analyzed using the GenomePAM pipeline with the off-target identification steps adapted from the GUIDE-seq pipeline (https://github.com/tsailabSJ/guideseq). The off targets were identified using the criteria of ≤ 6 mismatch bases with the intended targeting protospacer.

### GenomePAM Table

To identify enriched PAM motifs over genomic background, we develop an algorithm GenomePAM Table^67^, involving the computational steps implemented in a R script to: (1) Identify the most significantly enriched single-base motif: We define the edited value as the sum of GUIDE-seq detected genomic site numbers and GUIDE-seq read counts, with the latter linearly scaled to match the range of the former. The maximum value equals the highest number of genomic sites considered for all combinatorial potential motifs. Within the same motif window, a Chi-square test is used to compare the edited value against the corresponding genomic background counts among all motifs. (2) Extend from the position identified in Step 1 bidirectionally: Extend one base towards the 5′ end or one base towards the 3′ end and calculate the new edited values. Between the two extensions, the one with higher statistical significance is recorded and used for the next round of extension. (3) Repeat Step 2: Continue extending in both directions until the ends of candidate bases are reached. Record all significant motifs without limiting motif length. (4) Report enriched motifs: Report the enriched motifs along with the percentages of corresponding genomic sites edited, retaining only those motifs with increasing percentages from each iteration step (Fig. 1f).

## Data Availability

Details of target sites identified have been included in the Supplementary Tables. Raw Fastq data is available at SRA (ID 1258724 - BioProject - NCBI)^68^.
